# Insecticide resistance intensity in *Anopheles gambiae s.l.* populations from Nioro and Ndoffane health districts, Senegal

**DOI:** 10.1186/s12936-026-06067-9

**Published:** 2026-07-30

**Authors:** Souleymane Diallo, Moussa Diop, Mouhamadou Bassir Faye, Moussa Diallo, Abdoulaye Touré, Gnima Bodian, Ndeye Seny Diagne, Edouard Guedj Tine, Seynabou Mocote Diédhiou, Abdoulaye Niang, Lassana Konaté, Ibrahima Dia, El Hadji Amadou Niang

**Affiliations:** 1 Laboratoire d’Ecologie Vectorielle et Parasitaire, Faculté des Sciences et Techniques, Université Cheikh Anta Diop de Dakar, Dakar, Sénégal; 2 Institut National de Santé Publique de Guinée, Conakry, Guinée; 3 Pôle de Zoologie Médicale, Institut Pasteur de Dakar, Dakar, Sénégal

**Keywords:** *Anopheles gambiae s.l.*, Insecticide resistance, *kdr*, *Ace-1*, Resistance intensity, Senegal, Kaolack

## Abstract

**Background:**

Malaria remains a major public health concern in sub-Saharan Africa. In Senegal, the increasing resistance of *Anopheles gambiae *sensu lato (*s.l.*) to main public health insecticides threatens the effectiveness of long-lasting insecticide-treated nets (LLINs) and indoor residual spraying (IRS). This study aimed to assess insecticide resistance intensity and to determine the distribution of *kdr* and *Ace****-****1* resistance mutations in wild *An. gambiae s.l.* populations from Nioro and Ndoffane districts (Kaolack region).

**Methods:**

*Anopheles* larvae were collected between September and November 2018 and reared under insectary conditions. Adult females were subjected to World Health Organization (WHO) susceptibility assays using five insecticides: three pyrethroids, one carbamate and one organophosphate. Resistance intensity assays were conducted at 5 × and 10 × diagnostic concentrations. Species within the *An. gambiae* complex and the *East-kdr* (1014S)**,** *West-kdr* (1014F) and *Ace-1* (G119S) were identified using Polymerase Chain Reaction (PCR) technique.

**Results:**

A total of 1830 females *An. gambiae s.l.* were tested**.** Widespread resistance to pyrethroids and organophosphates was observed in both districts, while in Ndoffane complete susceptibility to bendiocarb was recorded and in Nioro resistance was observed. *Anopheles arabiensis* was the predominant species (> 90%), followed by *An. gambiae s.s.* and *An. coluzzii*. The frequencies of *East-kdr* and *West-kdr* mutations were higher in Ndoffane (40.4% and 30.8%) than in Nioro (10.6% and 13.1%). The *Ace-1* (G119S) mutation was detected exclusively in the heterozygous state across all the study populations.

**Conclusions:**

The studied vectors exhibit multiple insecticide resistance mechanisms, likely driven by combined selection pressures from agricultural and public health insecticide use. Strengthening entomological surveillance and implementing integrated resistance management strategies are essential to preserve the effectiveness of malaria vector control interventions in Senegal.

## Background

Malaria is a parasitic vector-borne disease transmitted to humans through the bites of infected female mosquitoes of the genus *Anopheles.* Despite the substantial progress in the control effort over recent decades, malaria remains one of the leading causes of morbidity and mortality worldwide [1]. Vector control strategies aim to reduce human-vector contact, vector density or lifespan in order to prevent transmission of *Plasmodium* parasites. The widespread deployment of long-lasting insecticide-treated nets (LLINs) and indoor residual spraying (IRS) has contributed significantly to the decline in malaria-related mortality and had raised realistic expectations its elimination by 2030. However, the emergence and rapid spread of insecticide resistance among main malaria vectors pose a serious threat to the sustainability of these achievements [2, 3]. Increasing insecticide use in agriculture, particularly for crop protection, has intensified selection pressure on mosquito populations, accelerating the development of resistance. Resistance to major insecticide classes, including pyrethroids, organochlorines and carbamates, has been widely reported across Africa [4, 5]. *Anopheles gambiae* populations have developed multiple resistance mechanisms [6]. These include metabolic resistance mediated by increased activity of detoxification enzymes such as cytochrome P450 mono-oxidases, esterases and glutathione-S-transferases as well as target-site resistance associated with single nucleotide polymorphism (SNP). The knock-down resistance (*kdr*) mutations *L1014F* (*West-kdr*) and *L1014S* (*East-kdr*) in the voltage-gated sodium channel gene are major contributors to the resistance to pyrethroids and DDT and are widely distributed in West and East Africa [7–9]. These mutations reduce insecticide binding to sodium channels, resulting in cross-resistance to pyrethroids and DDT. In addition, the G119S mutation in the acetylcholinesterase (*Ace-1*) gene is associated with the cross-resistance to carbamates and organophosphates [10, 11].

Effective vector control relies on the susceptibility of mosquito populations to the insecticides used under field conditions. However, laboratory bioassays increasingly reveal resistance in malaria vectors worldwide [12]. In Senegal, particularly within the Sudanian climatic zone, data on insecticide resistance intensity and the distribution of resistance mutations remain limited. Although previous studies have reported resistance to DDT and pyrethroids, comprehensive information on resistance mechanism and their contribution to phenotypic resistance is still insufficient [13–15].

In this context, National Malaria Control Programme (NMCP) of Senegal has implemented a national surveillance system to monitor insecticide resistance in malaria vectors. This study aimed to evaluate insecticide resistance intensity and to characterize resistance mechanisms in natural *An. gambiae* *s.l.* populations from Nioro and Ndoffane health districts in Kaolack region, in Central Senegal.

## Methods

### Study areas

The study was conducted in two health districts located in the Central Senegal, within the Kaolack region: namely in the health districts of Nioro (13°45' N, 15°48' W) and Ndoffane (13°56' N, 15°57' W). Field activities were carried out during the rainy season, from September to November 2018.

The Kaolack region belongs to the Sudano-Sahelian climatic zone, characterized by a single rainy season, from June to October, followed by a long dry season from November to May. In the Kaolack region, rainfall stations in Guinguinéo and Nioro recorded below-average rainfall compared to the 1981–2010 average (6 out of 10 stations in Nioro area). In addition, flooding events were reported in the commune of Keur Socé, where approximately 100 hectares of land were flooded between 3 and 9 September.

Both districts are predominantly agricultural, with extensive cultivation of groundnut and cotton, as well as subsistence crops such as millet, maize and sorghum. In recent years, off-season farming activities, including watermelon production and market gardening have expanded. Livestock farming also represents a major economic activity in the region, involving small ruminants, cattle, poultry, horses [16].

The study sites were selected based on their high anopheline diversity, which is closely linked to ecological heterogeneity. This diversity is supported by a dense hydrographic network and soil types favorable to the formation and persistence of a wide range of larval habitats. In addition, the sites were chosen within the framework of a comparative study between a former IRS area (Nioro district) and a control non-IRS area (Ndoffane district).

### Larval collection and rearing

**Fig. 1:**
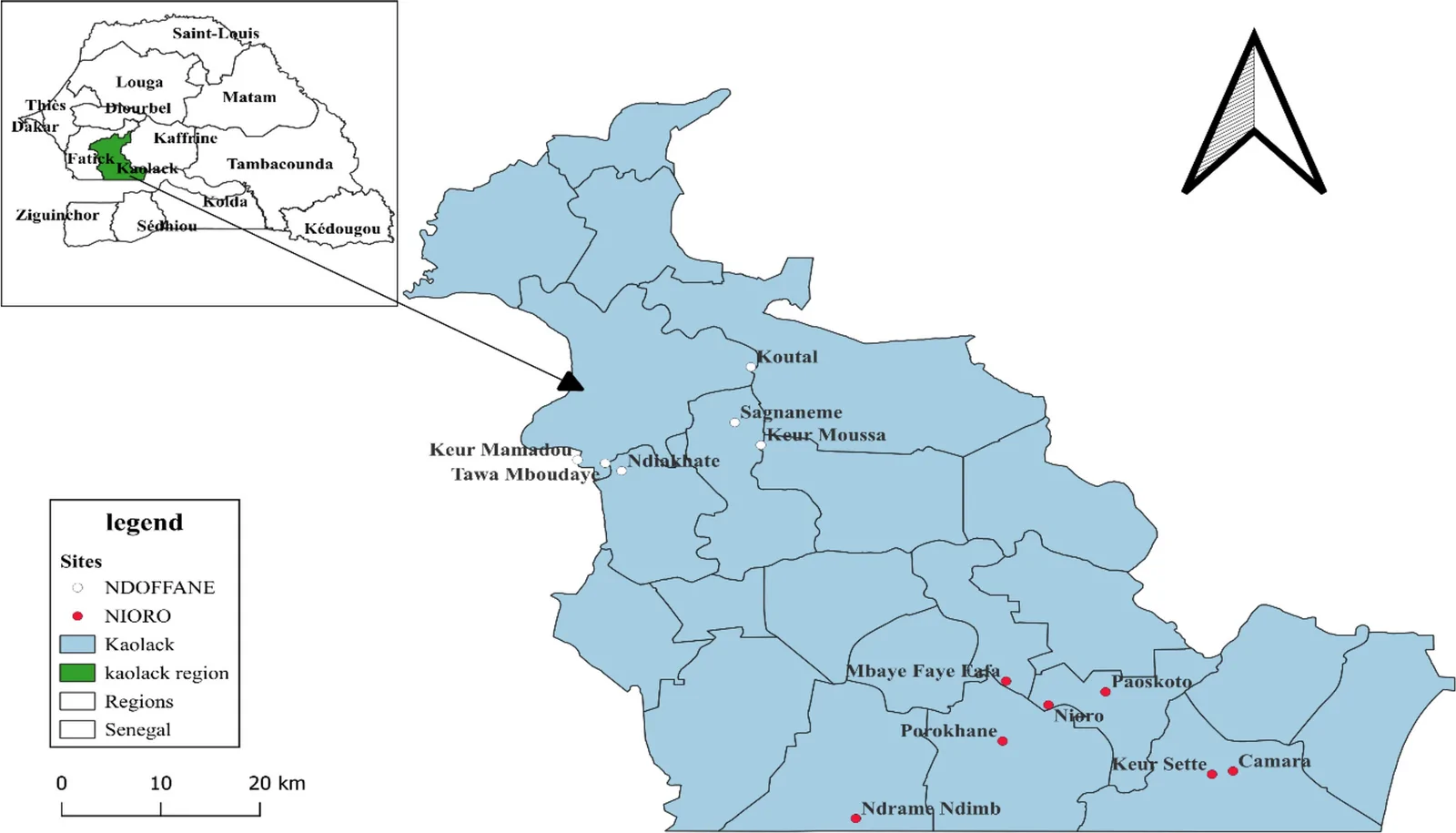
Map of the study sites within the Nioro and Ndoffance districts (Kaolack region, Senegal)

*Anopheles* larvae were collected from 13 villages across Nioro and Ndoffane health districts (Fig. 1) during the rainy season, from September to November 2018. In all study sites, long-lasting insecticide-treated nets constituted the primary malaria vector control intervention, with indoor residual spraying implemented in Nioro since 2007.

Immature stage of anopheline mosquitoes were collected using the dipping method and all larvae and pupae collected from positive breeding sites were transported and reared in the field insectarium. Larvae were fed with TetraMin® fish food. Emerged adults were fed a 10% sugar solution prior to insecticide susceptibility testing and maintained under standard insectarium conditions (temperature: 25 ± 2 °C and relative humidity: 80 ± 10%).

### WHO insecticide susceptibility tests

Insecticide susceptibility tests were conducted according to WHO guidelines [17], using 3–5 days old, non-blood fed female *An. gambiae s.l.* The susceptibility profiles of mosquito populations were assessed against five insecticides belonging to three chemical classes: pyrethroids (deltamethrin 0.05%, permethrin 0.75%, lambda-cyhalothrin 0.05%), a carbamate (bendiocarb 0.1%), and an organophosphate (pirimiphos-methyl 0.05%).

For each test, batches of 25 female mosquitoes were exposed for 60 min in WHO test tubes lined with insecticide-impregnated papers. Six tubes were used per assay: four containing insecticide-treated papers and two control tubes with solvent-impregnated papers. After exposure, mosquitoes were transferred to holding tubes with untreated paper and provided with 10% sugar solution and mortality was recorded 24 h post-exposure.

Resistance intensity assays were performed on additional samples of the same populations using insecticide concentrations 5 times and 10 times higher than the diagnostic doses, following WHO criteria.

### Species identification and detection of resistance mutations

After bioassays, both live and dead mosquitoes were processed for molecular analyses subsequently after being morphologically identified using the identification key described by Diagne et al. [18]. Molecular identification of sibling species within the *An. gambiae* complex was performed by PCR [19]. The knock-down resistance mutations L1014F (*West-kdr*) and L1014S (*East-kdr*) were detected using allele-specific PCR (IMP-PCR) [20]. Each PCR reaction contained 1–2 μL of CTAB-extracted DNA in a final volume of 23–25 μL, including Taq DNA polymerase, nuclease-free water, Green GoTaq^®^ 5X buffer, dNTPs (2.0–2.5 mM), MgCl_2_ (25 mM), and specific primers (2.5–25 pmol/μL).

Polymerase Chain Reactions were performed in a Bio-Rad^®^ thermal cycler under the following conditions: initial denaturation at 95 °C for 5 min, followed by 30 cycles (or 35 cycles for *kdr* assays) of denaturation at 95 °C for 30 s, annealing at 58 °C (or 57/59 °C for *East-kdr*/*West-kdr*) for 30 s, and 72 °C for 30 s, with a final extension at 72 °C for 5 min. Polymerase Chain Reaction products were separated on 2% agarose gels stained with Gel Red^®^ and visualized under UV light using a Bio-Rad^®^Gel Doc XR system.

The *Ace-1R* (G119S) mutation was detected by PCR in a total reaction volume of 21 µL containing ultrapure water, 5 × buffer, dNTPs, MgCl₂, specific primers, Taq polymerase, and DNA template.

### Data interpretation and statistical analysis

Data were recorded on a Microsoft Excel file and analyzed using WHO criteria for insecticide susceptibility test [21]. Bioassays were considered valid when control mortality was less than 5%, invalid when greater than 20%, and corrected using the Abbott formula when control ranged between 5 and 20% [22].

Mortality rates, genotypes, and allele frequencies were estimated for each population with 95% confidence intervals. The method for calculating the confidence interval for a binomial proportion was used to determine confidence interval. The Pearson’s chi-square test was used to compare *East-kdr* and *West-kdr* mutation frequencies. Allelic association studies were conducted using Fisher exact test and odds ratio test. Homogeneity of mortality rates was assessed using chi-square tests with a 5% threshold. All statistical analyses and graphical representations were performed using R software version 3.4.1 [23].

## Results

A total of 1830 female *An. gambiae s.l.* were tested against 5 insecticide molecules across both districts.

### Insecticides susceptibility

**Fig. 2:**
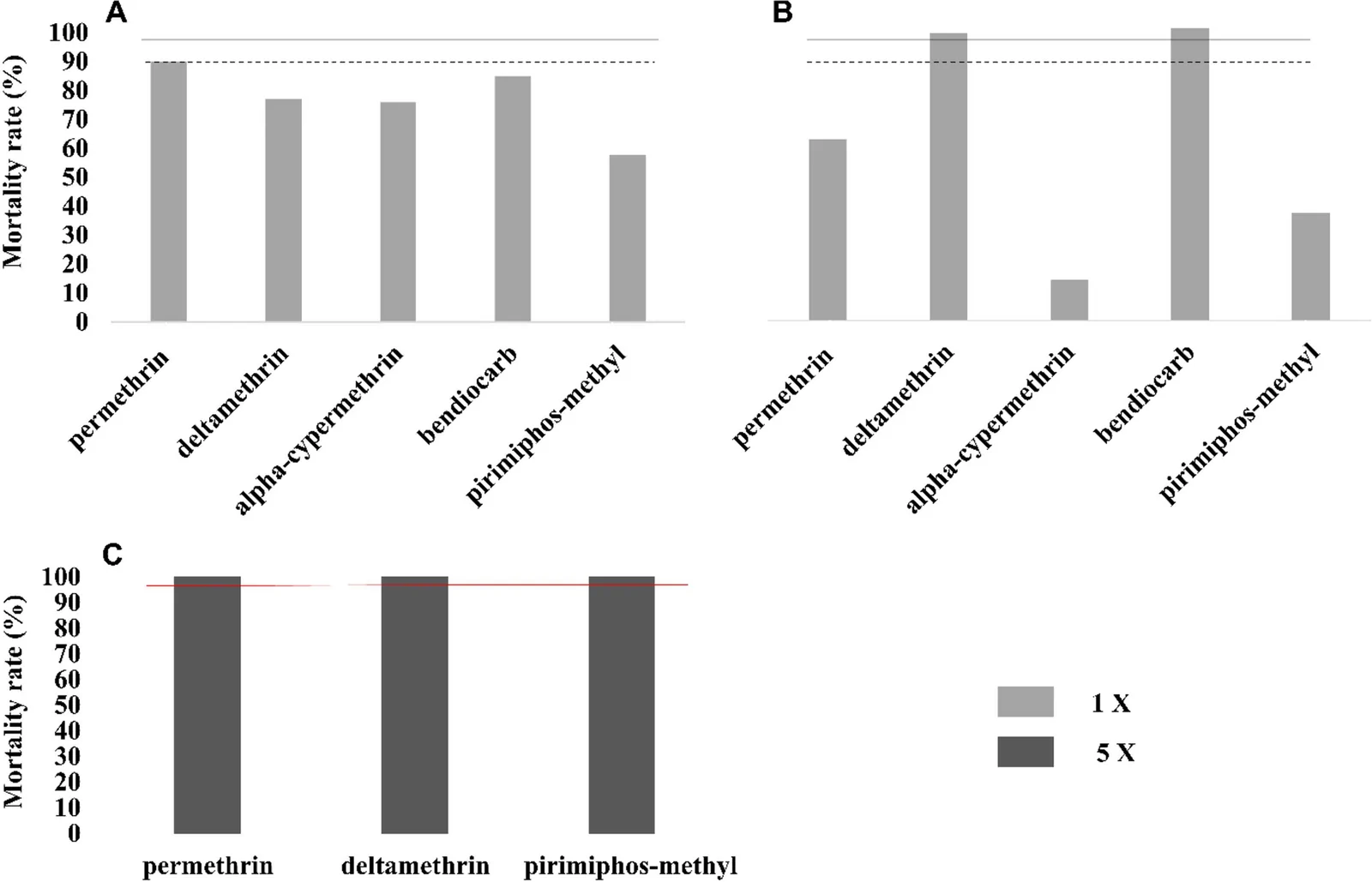
Post-exposure (24 h) mortality rates of *An. gambiae s.l.* to pyrethroids, bendiocarb and pirimiphos-methyl. **A** Nioro 1x. **B** Ndoffane 1x. **C** Nioro 5x. Solid and dotted horizontal bars indicate, respectively, susceptibility (98%) and resistance (90%) thresholds. Red bar indicates low intensity resistance (≥ 98%) and moderate to high intensity resistance (< 98%) threshold according to WHO criteria

In Ndoffane, standard WHO bioassays showed full susceptibility to bendiocarb (100% mortality; 95% CI 96.8–100), while resistance to pirimiphos-methyl (36.8% mortality; 95% CI 27–47.4) was observed. Regarding to pyrethroids, resistance was observed for both permethrin (62.0% mortality rate; 95% CI 52.1–71.2) and alpha-cypermethrin (13.9% mortality rate; 95% CI 7.9–21.8), whereas tested populations were susceptible to deltamethrin (98.2% mortality rate; 95% CI 78.5–99.7) (Fig. 2B).

In Nioro, resistance was confirmed to deltamethrin (77% mortality rate; 95% CI 70.5–86.9), alpha-cypermethrin (75.9% mortality rate; 95% CI 69.7–85.9), bendiocarb (85% mortality rate; 95% CI 77.3–90.8), pirimiphos-methyl (57.7% mortality rate; 95% CI 51.4–70.9) and possible resistance was observed to permethrin (90% mortality rate; 95% CI 83.9–95.8) (Fig. 2A).

### Resistance intensity

The resistance intensity assessed for permethrin at 5 × and 10 × in Ndoffane showed mortality rates of 97% and 99.1%, indicating moderate to high and moderate resistance respectively. In Nioro, resistance intensity to 5 × permethrin, 5 × deltamethrin, and 5 × pirimiphos-methyl was low to moderate, with mortality rates of 100% (Fig. 2C).

### Species composition

A total of 153 specimens were molecularly identified by PCR, including 73 from Ndoffane and 80 from Nioro (Table 1). In Ndoffane, *An. arabiensis* was the most predominant species, with 97.26% (n = 71), co-existing with both *An. gambiae* and *An. coluzzii,* each found with only a single specimen (1.37%, n = 1). A similar pattern was observed in Nioro, where *An. arabiensis* represented 90% (n = 72), while *An. gambiae* and *An. coluzzii* each accounted for 5% (n = 4).

**Table 1:** Genotypic and allelic frequencies of *East-kdr* (Vgsc-1014S) and *West-kdr* (Vgsc-1014F) mutations among the wild *An. gambiae s.l.* populations from Ndoffane and Nioro

Locations	Tested	n	Species	Genotypes				Alleles		
				SS	SRw	SRe	RwRe	S	Rw	Re
Ndoffane		71	*An. arabiensis*	(11) 15.49%	(1) 1.41%	(16) 22.54%	(43) 60.56%	(39) 27.46%	(44) 30.99%	(59) 41.55%
		1	*An. coluzzii*	(0) 0%	(1) 100%	(0) 0%	(0) 0%	(1) 50%	(1) 50%	(0) 0%
		1	*An. gambiae*	(1) 100%	(0) 0%	(0) 0%	(0) 0%	(2) 100%	(0) 0%	(0) 0%
	Total	73		(12) 16.44%	(2) 2.74%	(16) 21.92%	(43) 58.90%	(42) 28.77%	(45) 30.82%	(59) 40.41%
Nioro		72	*An. arabiensis*	(51) 70.83%	(4) 5.56%	(1) 1.39%	(16) 22.22%	(107) 74.31%	(20) 13.89%	(17)11.81%
		4	*An. coluzzii*	(4) 100%	(0) 0%	(0) 0%	(0) 0%	(8) 100%	(0) 0%	(0) 0%
		4	*An. gambiae*	(3) 75%	(1) 25%	(0) 0%	(0) 0%	(7) 87.50%	(1) 12.50%	(0) 0%
	Total	80		(58) 72.5%	(5) 6.25%	(1) 1.25%	(16) 20%	(122) 76.25%	(21) 13.13%	(17) 10.62%

*SS* homozygous susceptible, *SRw and SRe* heterozygous resistant, *RwRe* homozygous resistant, *n* number, Values indicate genotypic and allelic frequencies and values in parenthesis indicate the number of genotypes and alleles

### Genotypic and allelic frequencies of *East-kdr* and *West-kdr* mutations

Both the *East-kdr* (Vgsc-1014S) and the *West-kdr* (Vgsc-1014F) mutations, associated with the pyrethroid-DDT cross-resistance, were detected in both districts. In Ndoffane, the allele frequencies were 40.41% for Vgsc-1014S and 30.82% for Vgsc-1014F and were significantly higher than in Nioro, their frequencies were respectively 10.62% and 13.13% Vgsc-1014S: *p* = 2.21e-16; Vgsc-1014F: *p* = 7.19e-16).

Within each district, no statistically significant difference was observed between the frequencies of the two *kdr* mutations (Ndoffane: *p* = 0.15; Nioro: *p* = 0.56) (Table 1).

### Correlation between *kdr* alleles (*West-kdr*, *East-kdr*) and resistance phenotype to pyrethroids

Assessing the involvement of *kdr* mutation in *An. gambiae s.l.* susceptibility an allelic association analysis was performed on 80 specimens in Nioro and 73 specimens in Ndoffane including dead and alive mosquitoes exposed to pyrethroids. In Nioro, no significant correlations were observed between the both *West-kdr* (OR = 0.59 [0.23–1.52]; *p* = 0.28) and *East-kdr* alleles (OR = 0.78 [0.289–2.19]; *p* = 0.63) and the resistance phenotype to pyrethroids. Conversely, significant correlations were observed between the both *West-kdr* (OR = 3.06 [1.22–7.69]; *p* = 0.017) and *East-kdr* alleles (OR = 2.52 [1.05–6.06]; *p* = 0.039) and the resistance phenotype to pyrethroids in Ndoffane (Table 2).

**Table 2:** Association of *kdr* alleles with pyrethroid resistance phenotype in *An. gambiae s.l.* in Nioro and Ndoffane

Sites	Status	Genotypes				S/Rw		S/Re	
		SS	SRw	SRe	RwRe	OR [CI_95%_]	p-value	OR [CI_95%_]	p-value
Nioro	Dead	20	3	0	7	0.59 [0.23–1.52]	0.28	0.78 [0.28–2.19]	0.63
	Alive	38	2	1	9				
Ndoffane	Dead	9	2	12	21	3.06 [1.22–7.69]	0.017	2.52 [1.05–6.06]	0.039
	Alive	3	0	4	22				

*SS* homozygous susceptible, *SRw and SRe* heterozygous resistant, *RwRe* homozygous resistant, *S* susceptible allele, *Rw* west resistant allele, *Re* east resistant allele, *OR* odds ratio

### Genotypic and allelic frequencies of *Ace-1R* mutation

Overall, 105 specimens were screened for the *Ace-1R* mutation. In all positive specimens, the mutation was detected exclusively at the heterozygous (RS) state. In both Ndoffane and Nioro, the frequency of the *Ace-1R* mutation was higher in *An*. *arabiensis* than in *An*. *gambiae* and *An. coluzzii* (Table 3).

**Table 3:** Genotypic and allelic frequencies of the *Ace-1* mutation among the wild *An. gambiae s.l.* populations from Ndoffane and Nioro

Locations	Tested	Species	Genotypes		Alleles	
			SS	RS	S	R
Ndoffane	44	*An. arabiensis*	(18) 40.91%	(26) 59.09%	(62) 70.45%	(26) 29.55%
Nioro	53	*An. arabiensis*	(24) 45.28%	(29) 54.72%	(77) 72.64%	(29) 27.36%
	4	*An. coluzzii*	(2) 0.5%	(2) (0.5%)	(6) 7.5%	(2) 2.5%
	4	*An. gambiae*	(2) (0.5%)	(2) (0.5%)	(6) 7.5%	(2) 2.5%

*SS* homozygous susceptible, *SR* heterozygous resistant; Values indicate genotypic and allelic frequencies and values in parenthesis indicate the number of genotypes and alleles

## Discussion

This study provides updated evidence of widespread insecticide resistance among natural *An*. *gambiae s.l.* populations from Nioro and Ndoffane studied health districts in Central Senegal. More specifically, all populations exhibited resistance to pyrethroids, with the notable exception for the deltamethrin in Ndoffane, where the tested populations were susceptible. Resistance to pirimiphos-methyl was also detected in both districts as previously reported from Senegal and several other West African countries, including studies in Dakar (Bambilor) and Côte Ivoire (Agboville, Bouaflé, Divo, Tiassalé etc.) [24, 25]. Similarly, susceptibility to bendiocarb observed in Ndoffane has been reported in certain areas of Senegal and Benin [26–28], highlighting spatial heterogeneity in resistance profiles to this chemical.

In contrast, the study population of *An. gambiae s.l.* in Nioro displayed resistance to all the molecules tested, including pirimiphos-methyl and bendiocarb. This finding contrasts with earlier studies conducted shortly after the 2015 indoor insecticide spraying campaign [29], as well as in 2016 and 2017 as reported by PMI. The emergence of resistance to carbamates and organophosphates in this area suggests a rapid shift in resistance dynamics, likely driven by sustained selection pressure following the shift of IRS campaigns from pyrethroids to bendiocarb to pirimiphos-methyl. The resistance patterns observed in both districts may be attributed to the intensive and prolonged use of these insecticides both in the public health and agricultural settings. The widespread application of pyrethroids for vector control, combined with the use of organophosphates and carbamates such as dimethoate (DIMETO 400) for crop protection, particularly in cotton farming and market gardening, likely contributes to insecticide selection pressure on wild mosquito populations. In Nioro, repeated IRS campaigns may have further accelerated the selection of resistant phenotypes. Moreover, recent studies have highlighted that mosquito populations exposed to strong insecticide pressure may undergo physiological and behavioural adaptations allowing them to evade lethal doses, thereby exacerbating the development of the resistance [30]. This study represents the first assessment of insecticide resistance intensity in these two districts. Moderate to high resistance to permethrin was detected in Ndoffane, while resistance intensity in Nioro was generally low to moderate for permethrin, deltamethrin and pirimiphos-methyl. These findings are consistent with reports Nigeria, where similar resistance intensities to pyrethroids have been documented. The low resistance intensity observed for pirimiphos-methyl may reflect cross-resistance mechanisms shared with carbamates, given their common target site, acetylcholinesterase.

Three species of *An. gambiae* complex*,* namely *An. arabiensis*, *An. gambiae s.s.* and *An. coluzzii* were identified in both districts, with *An. arabiensis* being the most common species. This predominance is consistent with previous studies in Senegal, particularly in rural and semi-arid settings, where *An. arabiensis* is known to be ecologically advantaged due to its behavioural plasticity and tolerance to environmental variability and drought [31–33].

Molecular analysis revealed the presence of both *East-kdr* and *West-kdr* mutations in all the three species, confirming their involvement in the pyrethroid resistance. In Ndoffane, the *East-kdr* mutation was more frequent, consistent with earlier studies in the region of Dakar [34–37]. Conversely, the *West-kdr* predominated in Nioro. Notably, a substantial proportion of mosquitoes carried both mutations, suggesting ongoing recombination and complex resistance genotypes within local populations. Such co-occurrence has been widely reported across Africa and may enhance resistance levels or broaden resistance spectra [38].

The *Ace-1* mutation was also detected in both districts, exclusively at the heterozygous state. Although the frequency of the susceptible allele remained higher, the relatively elevated frequency of the resistant allele in *An. arabiensis* is of particular concern. The presence of the resistant allele in the different populations may explain the reduced efficacy of bendiocarb and pirimiphos-methyl observed in this study. Variations in *Ace-1* resistant allele frequency, likely reflecting differences in local insecticide selection pressures across habitats [37].

Genotypic analysis revealed contrasting resistance profiles between Ndoffane and Nioro, suggesting differentiated selection pressures, likely related to the history of indoor residual spraying (IRS) campaigns in the Nioro area. The *kdr* mutation contributes significantly to pyrethroid resistance, particularly in Ndoffane, although its correlation with the survival phenotype was not systematic. The presence of survivors with susceptible genotypes in Nioro, and to a lesser extent in Ndoffane, highlights the increasing role of metabolic mechanisms in the observed resistance. Furthermore, the complete absence of the RR genotype at the *Ace-1* locus in both sites suggests that resistance to organophosphates and carbamates is primarily driven by metabolic mechanisms rather than target-site modification. The predominance of metabolic resistance in Nioro, evidenced by the survival of individuals with susceptible genotypes at diagnostic doses, constitutes a major warning signal for resistance management. A study conducted in Côte d'Ivoire in 2023 reported overexpression of cytochrome P450 genes (notably *CYP6M2*, *CYP6P3* and *CYP9K1*), supporting the pyrethroid resistance observed in local malaria vectors [37]. Finally, our findings are partially consistent with those of a study carried out between 2013 and 2014 in Senegal, which included the Nioro area [38].

In terms of limitations, molecular analysis was restricted to a subsample of 153 specimens due to reagent constraints and the multi-site study design, which may limit statistical power. However, the allele frequencies observed were consistent across sites. The absence of synergist assays (e.g., with PBO) prevented us from fully characterizing the metabolic mechanisms involved. Finally, this study was conducted in 2018 with the aim of providing updated data on insecticide resistance levels in several localities in Senegal. Of note, a retrospective study published in 2021 traced the evolutionary history of DDT/pyrethroid cross-resistance in Senegal, reflecting the temporal dynamics of this phenomenon and underscoring the importance of continuous surveillance [15].

## Conclusions

This study updates the insecticide susceptibility and resistance intensity of *An. gambiae s.l.* populations in Nioro and Ndoffane health districts of Central Senegal. Widespread resistance to pyrethroids, carbamates and organophosphates was detected, with partial susceptibility to bendiocarb and deltamethrin, persisting in Ndoffane.

Resistance intensity was moderate in Ndoffane and low in Nioro. Molecular analyses revealed the presence of *Ace-1R*, *West-kdr* and *East-kdr* mutations**,** with contrasting spatial distributions. These findings highlight the need for strengthened resistance surveillance and the integration of synergist-based assays to inform adaptive vector control strategies.

## Data Availability

The datasets used and/or analysed during the current study are available from the corresponding author on reasonable request.

## Abbreviations

ACT: Artemisinin-based combination therapy
*An*.: *Anopheles*
CTAB: Cetyl-trimethyl ammonium bromide
DDT: Dichloro-diphenyl-trichloroethane
DNA: Desoxyribo-nucleic acid
DT: Diagnostic time
GSTs: Glutathione-S-Transferases
IMPs: Intentional mismatch primers
IRS: Indoor residual spraying
*kdr*: Knock-down resistance
LLINs: Long-lasting insecticide-treated nets
NMCP: National Malaria Control Programme
P450s: Cytochrome P450 monooxygenases
PBO: Piperonyl butoxide
PCR: Polymerase chain reaction
*s.l*: sensu lato
SNP: Single nucleotide polymorphism
*s.s.*: sensu stricto
VGSC: Voltage-gated sodium channel
WHO: World Health Organization

## References

[1] WHO. World malaria report 2025: addressing the threat of antimalarial drug resistance. Geneva: World Health Organization; 2025. p. 1–212.

[2] Tene-Fossog B, Fotso-Toguem YG, Amvongo-Adjia N, Ranson H, Wondji CS. Temporal variation of high-level pyrethroid resistance in the major malaria vector *Anopheles gambiae s.l.* in Yaoundé, Cameroon, is mediated by target-site and metabolic resistance. Med Vet Entomol. 2022;36:247–59. 10.1111/mve.12577 PMC9545389 35521949

[3] Reid MC, McKenzie FE. The contribution of agricultural insecticide use to increasing insecticide resistance in African malaria vectors. Malar J. 2016;15:107. 10.1186/s12936-016-1162-4 PMC4759738 26895980

[4] Oyewole IO, Mustapha K, Adedeji OC, Adeogun D, Awolola S. Susceptibility pattern of *Anopheles* mosquito to different classes of insecticides in selected communities in Ila-Orangun, Southwest Nigeria. Int J Mosq Res. 2018;5:106–11.

[5] Olatunbosun-Oduola A, Abba E, Adelaja O, Taiwo-Ande A, Poloma-Yoriyo K, Samson-Awolola T. Widespread report of multiple insecticide resistance in *Anopheles**gambiae**s*.*l*. mosquitoes in eight communities in Southern Gombe, North-Eastern Nigeria. J Arthropod-Borne Dis. 2019;13:50–61. PMC6643017 31346535

[6] Namountougou M, Simard F, Baldet T, Diabaté A, Ouédraogo JB, Martin T, et al. Multiple insecticide resistance in *Anopheles**gambiae**s*.*l*. populations from Burkina Faso, West Africa. Plos ONE. 2012;7:e48412. 10.1371/journal.pone.0048412 PMC3506617 23189131

[7] Ranson H, Jensen B, Vulule JM, Wang X, Hemingway J, Collins FH. Identification of a point mutation in the voltage-gated sodium channel gene of Kenyan *Anopheles gambiae* associated with resistance to DDT and pyrethroids. Insect Mol Biol. 2000;9:491–7. 10.1046/j.1365-2583.2000.00209.x 11029667

[8] Niang EHA, Konaté L, Diallo M, Faye O, Dia I. Patterns of insecticide resistance and knock down resistance (kdr) in malaria vectors *An*. *arabiensis*, *An*. *coluzzii* and *An*. *gambiae* from sympatric areas in Senegal. Parasit Vectors. 2016;9:71. 10.1186/s13071-016-1354-3 PMC4743422 26846990

[9] Keita K, Camara D, Barry Y, Ossè R, Wang L, Sylla M, et al. Species identification and resistance status of *Anopheles**gambiae**s*.*l*. (Diptera: Culicidae) mosquitoes in Guinea. J Med Entomol. 2017;54:677–81. 10.1093/jme/tjw228 28399224

[10] Essandoh J, Yawson AE, Weetman D. Acetylcholinesterase (*Ace-1*) target site mutation 119S is strongly diagnostic of carbamate and organophosphate resistance in *Anopheles gambiae s.s.* and *Anopheles coluzzii* across southern Ghana. Malar J. 2013;12:404. 10.1186/1475-2875-12-404 PMC3842805 24206629

[11] Keïta M, Kané F, Thiero O, Traoré B, Zeukeng F, Sodio AB, et al. Acetyl cholinesterase (*Ace-1R*) target site mutation G119S and resistance to carbamates in *Anopheles gambiae* (*sensu lato*) populations from Mali. Parasit Vectors. 2020;13:283. 10.1186/s13071-020-04150-x PMC7275337 32503614

[12] Djogbénou L. Malaria vector control and vector resistance to insecticides in Africa. Med Trop. 2009;69:160–4. 19545042

[13] Sabaly P, Ngom EHM, Gueye NA, Gueye A, Diallo M, Dia I. Differential insecticide resistance in *Anopheles arabiensis* populations in the seaside area of Mbour and its suburbs in Senegal. Heliyon. 2023;9:e21968. 10.1016/j.heliyon.2023.e21968 PMC10658326 38027803

[14] Gueye OK, Tchouakui M, Dia AK, Faye MB, Ahmed AA, Wondji MJ, et al. Insecticide resistance profiling of *Anopheles coluzzii* and *Anopheles gambiae* populations in the Southern Senegal: role of target sites and metabolic resistance mechanisms. Genes (Basel). 2020;11:1403. 10.3390/genes11121403 PMC7760107 33255805

[15] Diallo M, Hamid-Adiamoh M, Sy O, Sarr PC, Manneh J, Ndiath MO, et al. Evolution of the pyrethroids target-site resistance mechanisms in Senegal: early stage of the Vgsc-1014F and Vgsc-1014S allelic frequencies shift. Genes. 2021;12:1948. 10.3390/genes12121948 PMC8701854 34946897

[16] ANSD/SRSD. Kaolack: Regional Economic and Social Situation. Report. 2012;1–122.

[17] WHO. Procedures for testing insecticide resistance in malaria vector mosquitoes second edition. Geneva: World Health Organization; 2018.

[18] Diagne N, Fontenille D, Konate L, Faye O, Lamizana MT, Legros F, et al. The Anopheline of Senegal: an annotated and illustrated list. Bull Soc Path Exot. 1994;87:267–77. 7866049

[19] Wilkins EE, Howell PI, Benedict MQ. IMP PCR primers detect single nucleotide polymorphisms for *Anopheles gambiae* species identification, Mopti and Savanna rDNA types, and resistance to dieldrin in *Anopheles arabiensis*. Malar J. 2006;5:125. 10.1186/1475-2875-5-125 PMC1769388 17177993

[20] Huynh LY, Sandve SR, Hannan LM, Van Ert M, Gimnig JE. Fitness costs of pyrethroid insecticide resistance in *Anopheles gambiae*. In: Annual meeting of the society for the study of evolution, Christchurch, New Zealand. 2007.

[21] WHO. Procedures for testing insecticide resistance in mosquito vectors of malaria. Geneva: World Health Organization; 2017.

[22] Abbott WS. A method of computing the effectiveness of an insecticide. J Am Mosq Control Assoc. 1987;3:302–3. 3333059

[23] Karunarathne P, Pocquet N, Labbé P, Milesi P. BioRssay: An R package for analyses of bioassays and probit graphs. Parasit Vectors. 2022;15:35. 10.1186/s13071-021-05146-x PMC8785564 35073988

[24] Thiaw O, Doucouré S, Sougoufara S, Bouganali C, Konaté L, Diagne N, et al. Investigating insecticide resistance and knock-down resistance (*kdr*) mutation in Dielmo, Senegal, an area under long lasting insecticidal-treated nets universal coverage for 10 years. Malar J. 2018;17:123. 10.1186/s12936-018-2276-7 PMC5863856 29566682

[25] Camara S, Koffi AA, Ahoua-Alou LP, Koffi K, Kabran JK, Koné A, et al. Mapping insecticide resistance in *Anopheles**gambiae* (*s*.*l*.) from Côte d’Ivoire. Parasit Vectors. 2018;11:1–19. 10.1186/s13071-017-2546-1 PMC5759872 29310704

[26] Diop A, Konaté L, Faye O. Surveillance of the susceptibility of malaria vectors to insecticides. Annual report of the VBC project in Senegal. Dakar: Ministry of Health and Social Action; 2008.

[27] Diop A, Konaté L, Faye O. Surveillance of the susceptibility of malaria vectors to insecticides. Annual report of the VBC project in Senegal. Dakar: Ministry of Health and Social Action; 2010.

[28] Yadouleton WA, Padonou G, Asidi A, Moiroux N, Bio-Bangana S, Corbel V, et al. Insecticide resistance status in *Anopheles gambiae* in southern Benin. Malar J. 2010;9:83. 10.1186/1475-2875-9-83 PMC2858214 20334637

[29] Kwi PN, Dze JE, Tabue RN, Bigoga JD, Amambua-Ngwa A, Apinjoh TO. Distribution of *Anopheles**gambiae* s.l. siblings, insecticide resistance and prevalence of markers of resistance (Kdr & Ace-1) in Edea and Buea: forest region of Cameroon. BMC Infect Dis. 2025;25:864. 10.1186/s12879-025-11190-0 PMC12220066 40597727

[30] Robert V, Ayala D, Simard F. Medical and veterinary entomology. IRD Editions, Quae (229–230). 2017;218–19.

[31] Gatton ML, Chitnis N, Churcher T, Donnelly MJ, Ghani AC, Godfray HCJ, et al. The importance of mosquito behavioural adaptations to malaria control in Africa. Evolution. 2013;67:1218–30. 10.1111/evo.12063 PMC3655544 23550770

[32] Fontenille D, Lochouard L, Diatta M, Sokhna C, Dia I, Diagne N, et al. Four years’ entomological study of the transmission of seasonal malaria in Senegal and the bionomics of *Anopheles**gambiae* and *An*. *arabiensis*. Trans Roy Soc Trop Med Hyg. 1997;91:647–52. 10.1016/s0035-9203(97)90506-x 9509170

[33] Sougoufara S, Harry M, Doucouré S, Sembène PM, Sokhna C. Shift in species composition in the *Anopheles gambiae* complex after implementation of long-lasting insecticidal nets in Dielmo, Senegal. Med Vet Entomol. 2016;30:365–8. 10.1111/mve.12171 27058993

[34] Ndiath MO, Cailleau A, Orlandi-Pradines E, Bessell P, Pagès F, Trape JF, et al. Emerging knock-down resistance in *Anopheles arabiensis* populations of Dakar, Senegal: first evidence of a high prevalence of *kdr-east* mutation in West African urban area. Malar J. 2015;14:364. 10.1186/s12936-015-0898-6 PMC4579585 26395241

[35] Opondo KO, Jawara M, Cham S, Jatta E, Jarju L, Camara M, et al. Status of insecticide resistance in *Anopheles**gambiae* (*s*.*l*.) of The Gambia. Parasit Vectors. 2019;12:287. 10.1186/s13071-019-3538-0 PMC6549352 31164149

[36] Dia AK, Guèye OK, Niang EA, Diédhiou SM, Sy MD, Konaté A, et al. Insecticide resistance in *Anopheles arabiensis* populations from Dakar and its suburbs: role of target site and metabolic resistance mechanisms. Malar J. 2018;17:116. 10.1186/s12936-018-2269-6 PMC5856323 29544491

[37] Badolo A, Bando H, Traoré A, Ko-Ketsu M, Guelbeogo WM, Kanuka H, et al. Detection of G119S *Ace-1* ® mutation in field-collected *Anopheles gambiae* mosquitoes using allele-specific loop-mediated isothermal amplification (AS-LAMP) method. Malar J. 2015;14:477. 10.1186/s12936-015-0968-9 PMC4665897 26620269

[38] Diouf EH, Senghor MW, Sy MD, Ndiaye A, Konaté A, Niang O, et al. The impacts of indoor residual spraying with bendiocarb and pirimiphos-methyl on allelic frequencies of *kdr* and ace-1 mutations in central Senegal. Sci Rep. 2025;15:31239. 10.1038/s41598-025-12052-0 PMC12378222 40855152

